# Supplementary material for: A pitfall for machine learning methods aiming to predict across cell types
Source: Genome Biol. 2020 Nov 19;21:282. doi: 10.1186/s13059-020-02177-y (PMC7678316; doi:10.1186/s13059-020-02177-y)
Supplement: Supplementary file 1 — Additional file 1 Methods. Details related to the datasets used and the training and evaluation of the machine learning models presented in this work. [file 13059_2020_2177_MOESM1_ESM.pdf]

Additional File 1: Methods

Jacob Schreiber[1], Ritambhara Singh[2], Jeffrey Bilmes[1, 3], and William Stafford Noble*[1, 2]

[1]Paul G. Allen School of Computer Science & Engineering, University of Washington, Seattle, USA
[2]Department of Genome Science, University of Washington, Seattle, USA
[3]Department of Electrical & Computer Engineering, University of Washington, Seattle, USA

October 3, 2020

Data sets

Nucleotide sequence are extracted from the hg19 reference genome. Before input to our models, each sequence is one-hot encoded such that each genomic position is represented by four bits, of which only a single one is 1. For the task of active gene prediction, a 2 kbp region is extracted upstream of the transcription start site, accounting for the strand of the gene. For the task of TAD boundary prediction, a 2 kbp region is extracted from the middle of the 40 kbp region to be considered.

The ChIP-seq, DNase-seq and gene expression RPKM values were downloaded from the Roadmap compendium (https://egg2.wustl.edu/roadmap/data/byFileType/signal/consolidated/macs2signal/pval/ and https://egg2.wustl.edu/roadmap/data/byDataType/rna/expression/). Each ChIP-seq and DNase-seq experiment is reported using $-\log_{10}$ p-values, indicating the statistical significance of the enrichment of the measured phenomenon at each genomic position. Additionally, these tracks are *arcsinh* transformed, which is similar to a log transform and is a standard technique to reduce the effect of outliers on the model. After this transformation, the average signal value for each epigenomic mark across the 2 kbp region of interest is used as input to our models. We used experimental measurements of H3K4me3, H3K27me3, H3K36me3, H3K9me3, and H4K3me1 for the prediction of gene expression, and additionally measurements of DNase-seq and H3K27ac for predicting TAD boundaries.

Gene bodies were defined as GENCODE v19 gene elements (https://www.gencodegenes.org/human/release_19.html) on Chr1–22, resulting in 17,951 gene bodies for each of 56 different human cell types. We define active genes as those that have an RPKM value of > 0.5.

TAD boundary calls were obtained from the supplementary material of[1] for the seven cell lines TRO, H1, NPC, GM12878, MES, IMR-90, and MSC. These calls are binary indicators and were specified at 40 kbp resolution.

Predictions from the top four participants in the ENCODE-DREAM challenge and the CTCF test set labels were provided by the ENCODE-DREAM challenge organizers. The training set CTCF peak calls were downloaded from the challenge website. All data from the challenge is used with permission from the organizers.

Model architectures

We evaluated the performance of a variety of neural network models for our tasks. For models that used only epigenomic signal as input, we considered all models that had between 1 and 5 layers and all powers of 2 between 1 and 4096 neurons per layer.

*Corresponding author: william-noble@uw.edu

For models that used only nucleotide sequence as input, we considered two different types of models. The first are fully dense networks similar to those that used only epigenomic signal. These models had between 1 and 3 layers with all powers of 2 between 1 and 1024 neurons per layer. The second are convolutional models that are composed of a variable number of convolutional layers followed by max pooling layers and ending with a single dense layer. These convolutional models had between 1 and 3 convolutional layers, between 1 and 256 filters per convolutional layer, and between 1 and 1024 nodes in the final dense layer. The convolutional layers used a kernel of size 8 and a stride of 1. The max pooling layers had a kernel of size 4 and a stride of 4.

The models that used both nucleotide sequence and epigenomic signal were composed of one of the nucleotide models above and one of the epigenomic models. The final hidden layers of the two models were concatenated together and fed through an additional hidden layer before the output. Rather than consider all potential model architectures that utilized nucleotide sequence, we limited our evaluation to only 100 randomly selected model architectures for computational reasons.

In all models, both the convolutional layers and the hidden dense layers used ReLU activations, where $f(x) = max(0, x)$.

Model training

The neural network models were trained in a standard fashion for neural network optimization. This involved using the Adam optimizer[2] and a binary cross-entropy loss. All model hyperparameters were set to their defaults as specified by Keras version 2.0.8 (https://keras.io), and no additional regularization was used. The models were trained on balanced mini-batches of size 32, and an epoch was defined as 400 mini-batches. Training proceeded for 100 epochs, but was stopped early if performance on a balanced validation minibatch of size 3,200 did not improve after five consecutive epochs.

The gradient boosted decision tree models were trained using XGBoost[3]. The default values were used for all parameters, except that training progressed for 300 iterations, instead of 100, and the maximum depth of each tree was set to 6, instead of 3. The model was trained using a binary logistic loss and a L2 regularization strength of 1. A single model was trained for each input feature set. These models are then evaluated using the first N trees, using N between 1 and 300, to get the performance of models of varying complexity. Because subsampling is not used, this procedure is identical to independently training models of varying sizes.

The training, validation, and test sets consisted of different genomic loci depending on the model evaluation setting. In the cross-chromosomal setting, the validation set was derived from Chromosome 2 and the test set was derived from Chromosome 1 for both tasks. For the gene expression task, the training set consisted of all genes in Chromosomes 3 through 22, while for the TAD boundary prediction task, it consisted of all 40 kbp bins in Chromosome 3. In the cross-cell type setting, the training, validation, and test sets were derived from Chromosomes 2 through 22 in the gene expression task and Chromosomes 2 and 3 in the TAD boundary prediction task. In the hybrid setting, the training and validation sets were the same as in the cross-cell type setting, but the test set for both tasks were samples derived from Chromosome 1. We chose to hold the training set constant between the cross-cell type and hybrid approaches, rather than the test set, in order to demonstrate that models trained on the same data exhibit markedly different trends with respect to model complexity depending on the evaluation set.

Depending on the evaluation setting, these models were also trained on either a single, or multiple, cell types. In all cases, models were evaluated on data derived from the H1 cell line (E003). In the cross-chromosomal setting, models for both tasks were also trained on data from the H1 cell line (E003). For the gene expression task in both other settings, samples drawn from spleen (E113), H1 BMP4 derived mesendoderm cultured cells (E004), CD4 memory primary cells (E037), and sigmoid colon (E106) were used as the validation set, and all other cell types (excluding the H1 cell line) were used as the training set (see Additional file 1: Table S1). For predicting TAD boundaries, the validation set was drawn from GM12878 (E116) and the training set consisted of all other cell lines (excluding the H1 cell line).

Average activity

The average activity is the value of some form of biological signal averaged, at each genomic locus, over all cell types in the training set. Formally, for a training set with m cell types, n genomic loci, and measurements of some signal $S \in \mathbb{R}^{m,n}$, the average activity $A \in \mathbb{R}^n$ is calculated as

$$A_i = \frac{1}{m} \sum_{j=1}^{m} S_{j,i}$$

Average precision

For each task we evaluated model performance using the average precision score. Average precision refers to the average of the precision multiplied by the recall using each point as a threshold. The average precision is an estimate of the area under a precision-recall curve that is not overly optimistic, as is the case when one linearly interpolates between points in the curve. The score is calculated by

$$AP = \sum_{n} (\text{Recall}_n - \text{Recall}_{n-1}) \, \text{Precision}_n$$

where Recall_n and Precision_n are the recall and the precision at the n-th calculated threshold, with one threshold for each data point.

References

[1] Schmitt AD, Hu M, Jung I, Xu Z, Qiu Y, Tan CL, et al. A Compendium of Chromatin Contact Maps Reveals Spatially Active Regions in the Human Genome. Cell Reports. 2016;17:2042–2059.

[2] Kingma D, Ba J. Adam: A Method for Stochastic Optimization. In: Proceedings of the 3rd International Conference on Learning Representations; 2015. .

[3] Chen T, Guestrin C. XGBoost: A Scalable Tree Boosting System. In: Proceedings of the 22nd ACM SIGKDD International Conference on Knowledge Discovery and Data Mining. KDD '16. New York, NY, USA: ACM; 2016. p. 785–794.
