# Supplementary material for: A pitfall for machine learning methods aiming to predict across cell types
Source: Genome Biol. 2020 Nov 19;21:282. doi: 10.1186/s13059-020-02177-y (PMC7678316; doi:10.1186/s13059-020-02177-y)
Supplement: Supplementary file 2 — Additional file 2 The supplementary figures and tables references in this work. [file 13059_2020_2177_MOESM2_ESM.pdf]

# Additional File 2: Supplementary Figures and Table

Jacob Schreiber[1], Ritambhara Singh[2], Jeffrey Bilmes[1, 3], and William Stafford Noble*[1, 2]

[1]Paul G. Allen School of Computer Science & Engineering, University of Washington, Seattle, USA
[2]Department of Genome Science, University of Washington, Seattle, USA
[3]Department of Electrical & Computer Engineering, University of Washington, Seattle, USA

October 3, 2020

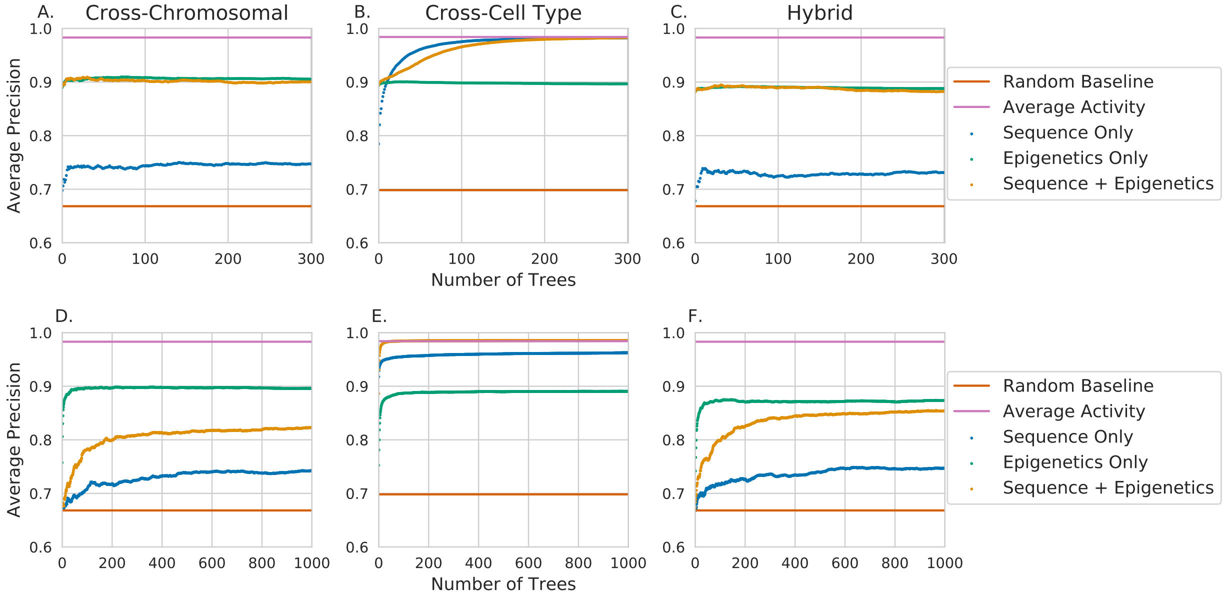


Fig. S1: **The performance of a gradient boosted decision tree classifier and a random forest classifier with a varying number of trees in three predictive settings for predicting gene expression.** The figure plots the average precision (AP) of a gradient boosted decision tree model predicting gene expression as a function of model complexity. Evaluation is performed via (a) cross-chromosome, (b) cross-cell type, and (c) a combination of cross-chromosome and cross-cell type validation. In each panel, each point represents the test set performance of a single trained model. (d-f) The same as a-c but using a random forest classifier instead of a gradient boosted decision tree classifier.

*Corresponding author: william-noble@uw.edu

| Roadmap ID | Biosample Summary |
|---|---|
| E004 | H1 BMP4 Derived Mesendoderm Cultured Cells |
| E005 | H1 BMP4 Derived Trophoblast Cultured Cells |
| E006 | H1 Derived Mesenchymal Stem Cells |
| E007 | H1 Derived Neuronal Progenitor Cultured Cells |
| E011 | hESC Derived CD184+ Endoderm Cultured Cells |
| E012 | hESC Derived CD56+ Ectoderm Cultured Cells |
| E013 | hESC Derived CD56+ Mesoderm Cultured Cells |
| E016 | HUES64 Cell Line |
| E024 | 4star |
| E027 | Breast Myoepithelial Cells |
| E028 | Breast vHMEC |
| E037 | CD4 Memory Primary Cells |
| E038 | CD4 Naive Primary Cells |
| E047 | CD8 Naive Primary Cells |
| E050 | Mobilized CD34 Primary Cells Female |
| E053 | Neurosphere Cultured Cells Cortex Derived |
| E054 | Neurosphere Cultured Cells Ganglionic Eminence Derived |
| E055 | Foreskin Fibroblast Primary Cells skin01 |
| E056 | Foreskin Fibroblast Primary Cells skin02 |
| E057 | Foreskin Keratinocyte Primary Cells skin02 |
| E058 | Foreskin Keratinocyte Primary Cells skin03 |
| E059 | Foreskin Melanocyte Primary Cells skin01 |
| E061 | Foreskin Melanocyte Primary Cells skin03 |
| E062 | Peripheral Blood Mononuclear Primary Cells |
| E065 | Aorta |
| E066 | Adult Liver |
| E070 | Brain Germinal Matrix |
| E071 | Brain Hippocampus Middle |
| E079 | Esophagus |
| E082 | Fetal Brain Female |
| E084 | Fetal Intestine Large |
| E085 | Fetal Intestine Small |
| E087 | Pancreatic Islets |
| E094 | Gastric |
| E095 | Left Ventricle |
| E096 | Lung |
| E097 | Ovary |
| E098 | Pancreas |
| E100 | Psoas Muscle |
| E104 | Right Atrium |
| E105 | Right Ventricle |
| E106 | Sigmoid Colon |
| E109 | Small Intestine |
| E112 | Thymus |
| E113 | Spleen |
| E114 | A549 EtOH 0.02pct Lung Carcinoma |
| E116 | GM12878 Lymphoblastoid |
| E117 | HeLa-S3 Cervical Carcinoma |
| E118 | HepG2 Hepatocellular Carcinoma |
| E119 | HMEC Mammary Epithelial |
| E120 | HSMM Skeletal Muscle Myoblasts |
| E122 | HUVEC Umbilical Vein Endothelial Cells |
| E123 | K562 Leukemia |
| E127 | NHEK-Epidermal Keratinocytes |
| E128 | NHLF Lung Fibroblasts |

Table S1: **Training cell lines used to predict gene expression in the cross-cell type and hybrid evaluation settings**. The Roadmap Epigenomics Mapping Consortium ID and biosample description are given for each.

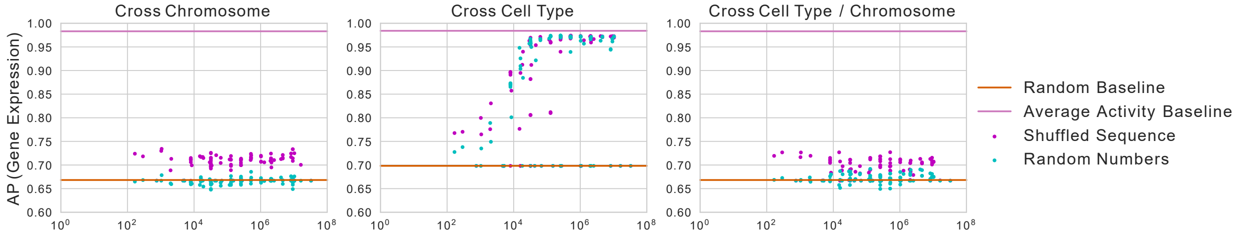


Fig. S2: **The performance of neural networks with randomized inputs.** The figure plots the average precision (AP) of neural network models predicting gene expression using randomized inputs as a function of model complexity. The inputs are either one-hot encoded nucleotide sequence that has been permuted (in cyan) or Gaussian random values (in purple). In both cases, the representation for each gene is consistent across cell types. Evaluation is performed via (a) cross-chromosome, (b) cross-cell type, and (c) a combination of cross-chromosome and cross-cell type validation. In each panel, each point represents the test set performance of a single trained model.

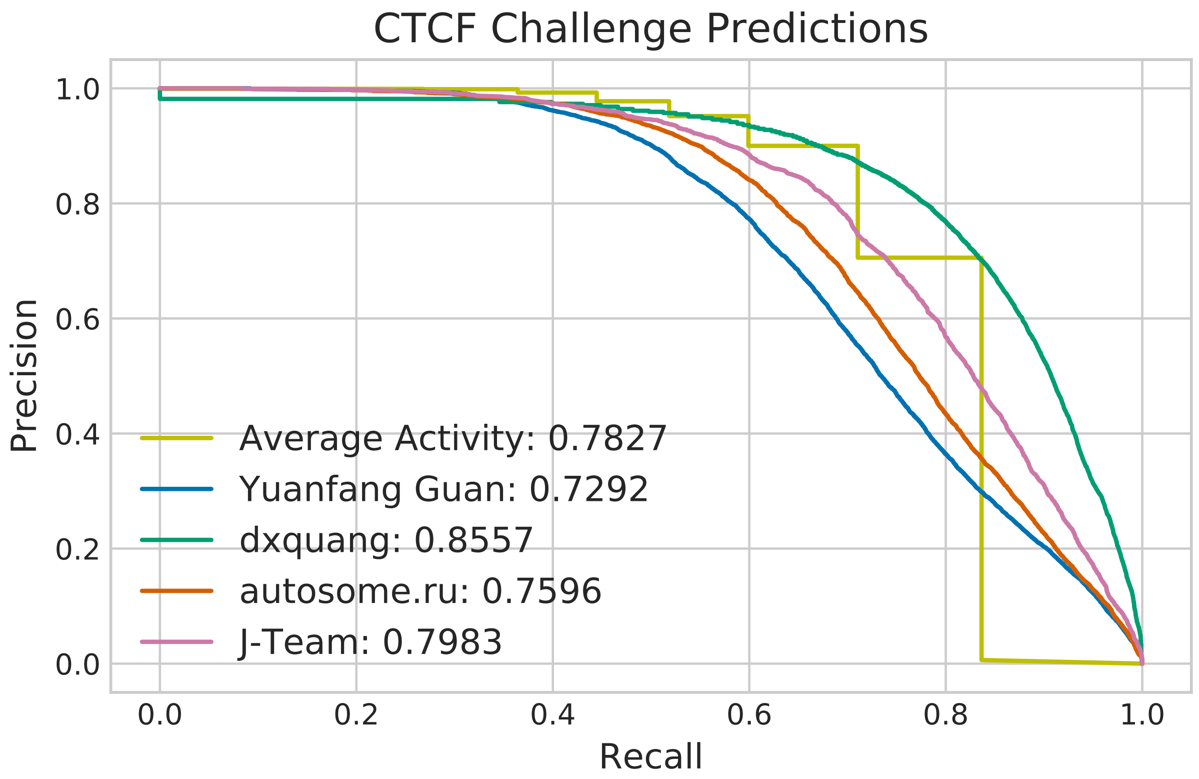


Fig. S3: **Performance of the top four participants in the ENCODE-DREAM TF binding challenge compared to the average activity baseline at predicting CTCF in iPSC.** Precision-recall curves for each of the top four participants in the ENCODE-DREAM TF binding challenge, as well as a precision-recall curve for the average activity baseline at the same task. The average precision of each approach is shown in the legend.

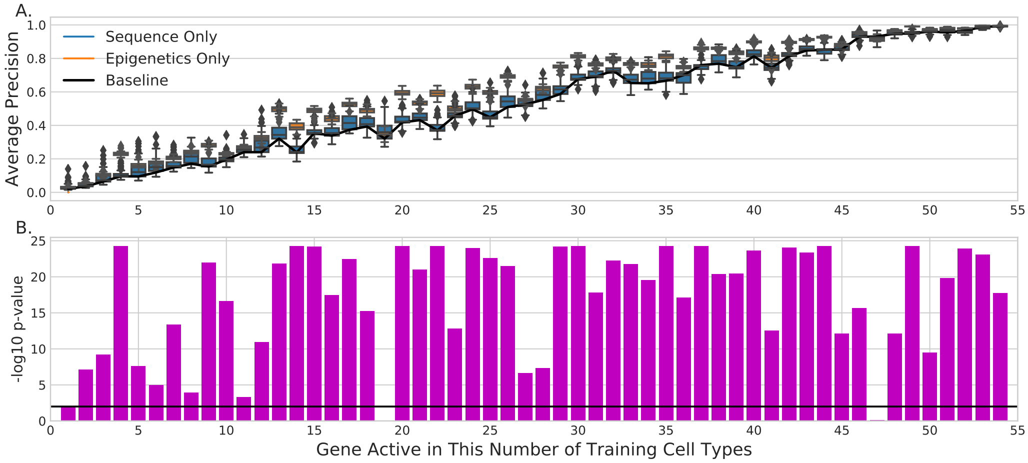


Fig. S4: **Epigenomic signal yields more predictive models than nucleotide sequence in the cross-cell type setting when locus-specific biases are factored out.** Genes in the cross-cell type setting were split into 54 groups based on the number of training and validation set cell types that they are active in. (a) For each group, the AP score was calculated using the predicted probabilities from models that use only nucleotide sequence or use only epigenomic signal. Each box shows the three quartile values, with whiskers extending to 1.5 the inter-quartile range. (b) The AP scores from those two groups were then compared using a one-sided Mann-Whitney U test. The -log10 p-values of this test are displayed for each group. The null hypothesis is rejected for most groups, indicating that models that use epigenomic signal outperform those that use only nucleotide sequence when the average activity is factored out of the evaluation. As expected, the epigenetics-only case is relatively better as the uncertainty increases, corresponding to the middle of the plot above.

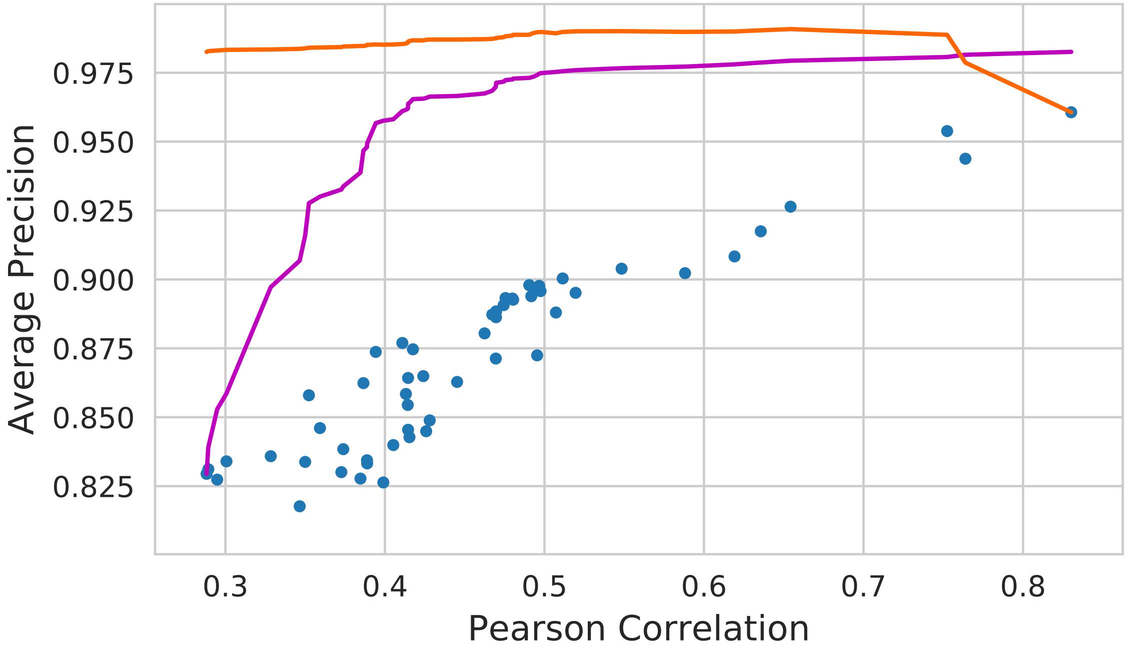


Fig. S5: **The performance of the average activity baseline as a function of cell type similarity.** The strength of the average activity baseline in the cross-cell type setting at predicting H1 gene expression when calculated using three different approaches. The first approach is to calculate it, not as an average over several cell types, but individually from each cell type in the training set (blue dots) where the average precision is plotted as the y-axis and the correlation with gene expression in H1 is the x-axis. The second approach is to average gene expression values across all cell types that are at least as similar as a certain cell type, i.e. average over all cell types to the right (orange line). The third approach is to average gene expression values over all cell types that are no more similar than a certain cell type, i.e. average over all cell types to the left (magenta).
